# SUBCUTANEOUS PANNICULITIS-LIKE T-CELL LYMPHOMA

**DOI:** 10.4103/0019-5154.70707

**Published:** 2010

**Authors:** Abel Francis, S Criton, Sandhya Acharya, Anitta Shojan, Rashmi Mary Philip

**Affiliations:** *From the Assistant Professor, Department of Dermatology, Amala Institute of Medical Sciences, (AIMS), Thrissur, Kerala, India*

**Keywords:** *Subcutaneous panniculitis-like*, *T-cell lymphoma*, *rimming of fat cells*

## Abstract

This case report describes a 38 year-old lady with the clinical, histopathological, and immunohistochemical (IHC) changes of subcutaneous panniculitis-like T-cell lymphoma (SPTCL). The IHC findings revealed CD8 + and CD56 – cells, which are indicative of tumors which have an indolent course. Our patient is being managed with tapering doses of corticosteroids for the last nine months with good improvement.

## Introduction

Subcutaneous panniculitis-like T-cell lymphoma (SPTCL) is a rare cutaneous T-cell lymphoma that resembles panniculitis. It was originally described by Gonzalez *et al*. in 1991.[1] It is clinically characterized by subcutaneous nodules and plaques, which usually involve the legs and less commonly, the trunk. Histopathological investigations reveal the presence of primarily subcutaneous infiltrates of small,, medium-sized, or large pleomorphic T cells and macrophages. These cases have an αβ T cell receptor (TCR) and are usually CD8 + and CD56 –.[2]

## Case Report

**Figure 1:**
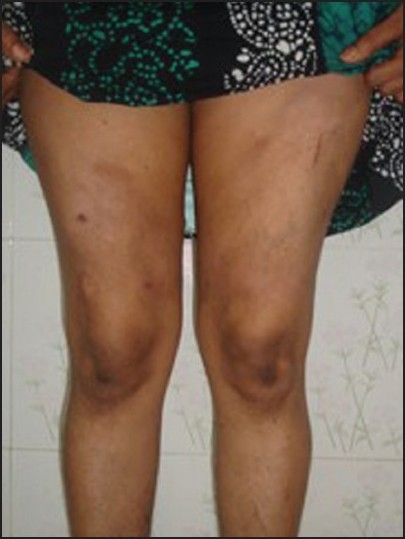
Nodule showing central atrophy and peripheral erythema and scaling

**Figure 2:**
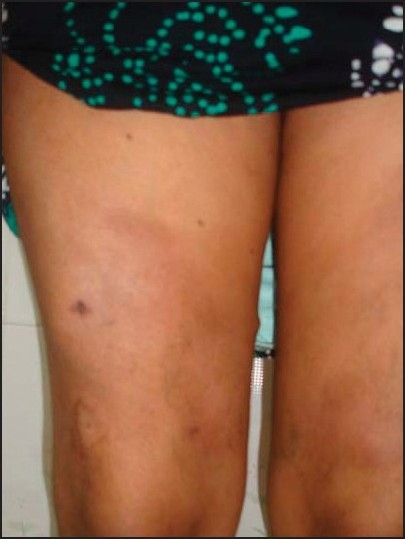
Close up view of nodule showing central atrophy and peripheral erythema and scaling

A 38 year-old lady who works as a cook, presented to our outpatient department with a 1 ½ years’ history of multiple painful nodules of varying sizes on both the thighs. There was no history of fever, fatigue, weight loss, or any systemic symptoms. Examination revealed that the nodules were indurated, tender, and with peripheral erythema; some of the nodules showed central atrophy with peripheral scaling [Figures 1 and 2]. Oral and genital mucosal tissues were normal. There were no nail or hair changes. General examination did not reveal any significant findings. There was no lymphadenopathy and systemic examination results were also normal. An earlier incision biopsy done eight months ago, suggested panniculitis with fat necrosis.

The results for hematological and biochemical investigations were as follows [Table 1].

**Table 1:** Investigations

Investigations	Value
TLC	6800/cu. mm
Hb	12.5 g/dL
Neutrophils	80%
Lymphocytes	20%
ESR	38 mm/1 h
Blood glucose random	94 mg/dL
Blood urea	20 mg/dL
S. creatinine	0.7 mg/dL
Calcium	9.0 mg/dL
Total protein	6.8 g/dL
Albumin	3.8 g/dL
Globin	3.0 g/dL
A/G	1.2 : 1
Bilirubin – total	1.3 mg/dL
Bilirubin – direct	0.3 mg/dL
AST (SGOT)	24 U/L
ALT (SGPT)	10 U/L
Alkaline phosphatase	51 U/L
LDH	510U/L
Serum amylase	56 U/L
ANA	Negative
Peripheral smear	WNL
Urinalysis	WNL

Chest X ray and ultrasound of the abdomen were within normal limits.

**Figure 3:**
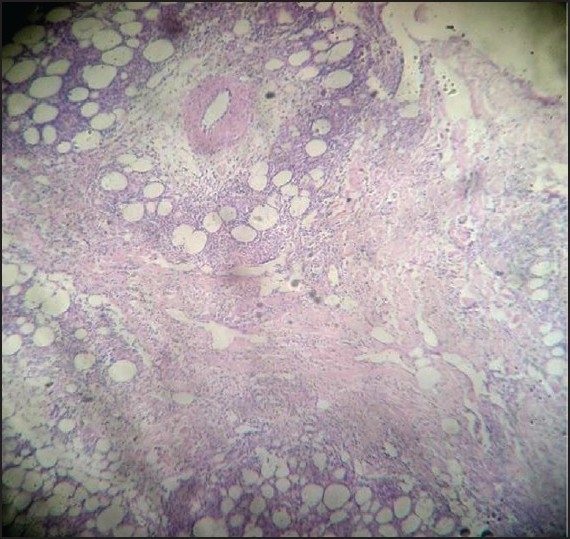
Scanner view showing panniculitis (H and E stain, ×40)

**Figure 4:**
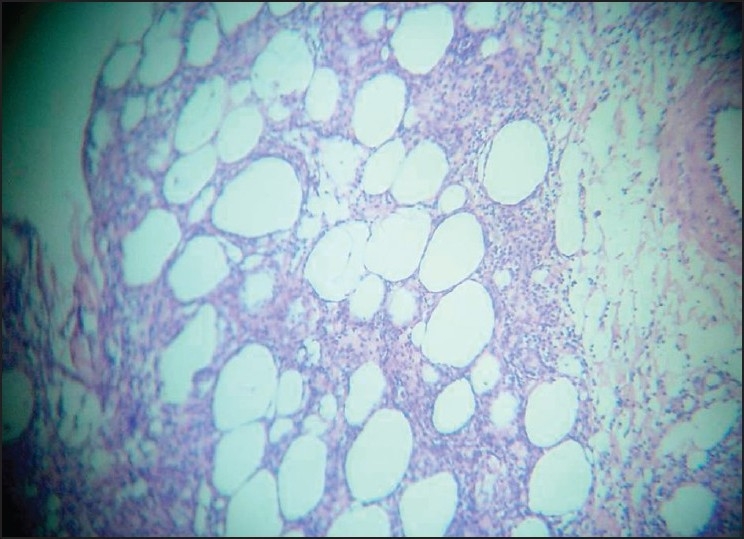
Low power showing panniculitis (H and E stain, ×100)

**Figure 5:**
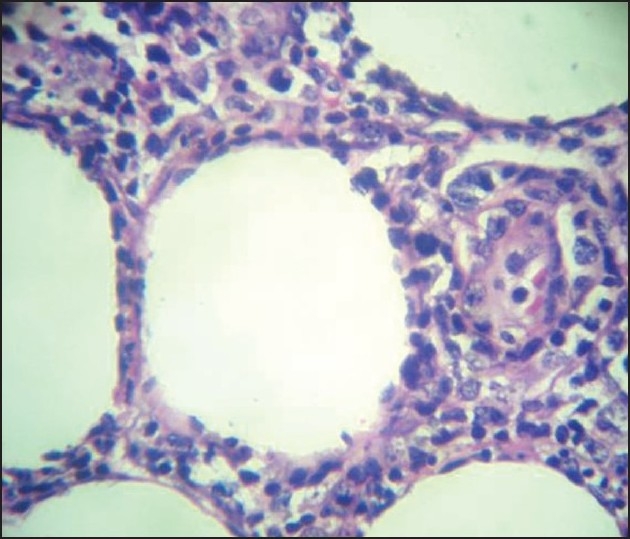
Rimming of fat cells by atypical lymphoid cells (H and E stain, ×100)

A repeat punch biopsy was done and the subcutis showed an infiltrate of small to medium-sized lymphoid cells that were pleomorphic and had hyperchromatic nuclei with karyorrhexis. There was rimming of some of the fat cells by atypical lymphoid cells. The upper dermis showed a mildly perivascular infiltrate of lymphocytes [Figures 3–5]. The epidermis was unremarkable. Immunohistochemical analysis showed that the cells were CD3 +, CD8 +, CD20 – and CD56 –; the Ki-67 index was 70–80%. The biopsy was sent to two other centers for confirmation of the diagnosis of SPTCL. Similar results were obtained and a review of the earlier biopsy also showed features suggestive of SPTCL.

The patient was referred to the Oncology Department where they suggested chemotherapy which she deferred. Hence, prednisolone 1 mg/kg body weight was started and the lesions became less indurated within a month. The steroid was then gradually tapered so that now, nine months after the diagnosis, she takes 5 mg prednisolone on alternate days. The patient is symptomatically better and no new lesions have appeared.

## Discussion

SPTCL is a rare T-cell lymphoma recognized by the new World Health Organization (WHO)-European Organization for Research and Treatment of Cancer (EORTC) classification for cutaneous lymphomas.[2] Lymphomas expressing TCR - αβ which is normally CD8 + and CD56 –, are restricted to the subcutaneous tissue and often run an indolent course. In contrast, cases with TCR γδ are CD4 –, CD8 – and CD56 + and may involve the epidermis and have a poor prognosis.[2–4] On this basis, according to the WHO-EORTC classification, SPTCL is restricted to cases with αβ TCR and those with TCR γδ are placed in a new category of primary cutaneous peripheral T cell lymphomas—cutaneous γ T-cell lymphoma.[2]

Each T cell is genetically programmed to recognize a specific cell-bound antigen by means of an antigen-specific T cell receptor. In approximately 95% of T cells, the TCR consists of a disulfide-linked heterodimer made up of an a and α β polypeptide chain, each having a variable and a constant region. In a minority of T cells, another type of TCR, composed of, γ and δ polypeptide chains is found. TCR diversity is generated by somatic rearrangement of the genes that encode the α, β, γ, and δ TCR chains.[5]

There are various reasons for missing a histopathologybased diagnosis. In the early stages of SPTCL, the infiltrate may lack significant atypia, and the classical histopathology may not be evident later when the lesions are regressing.[4] In our case, the diagnosis may have been initially missed because of the lack of familiarity of the general pathologist, who reported the first biopsy, with SPTCL.

Another important finding with our patient was that she was CD56 –. SPTCL patients who are CD56 – have been reported to have a better prognosis[2] due to which we went ahead with a very nonaggressive line of management.

According to the earlier classification, SPTCL was considered to be a very aggressive disease with a poor prognosis. This was because both tumors with TCR αβ as well as those with TCR γ were classified together as a single entity. Currently, tumors with TCR γδ are classified separately and SPTCL has TCR ab which is CD56 – and has a better prognosis than the other group which has TCR γδ and is CD56 +.

Evaluation of the degree of cellular proliferation of malignant cells can be done by immunohistochemical techniques using Ki-67 antigen that is expressed in the G1, S, G2 phases of the cell cycle (not in the G0 phase), the highest levels being found in the M phase. Its expression is associated with a high mitotic count and a high histology grade. When the Ki-67 index is higher than 20%, there is a shortened overall survival period and increased chance of development of metastasis.[6] Our patient had a Ki-67 index of 70–80% that warranted an aggressive line of therapy. However, our patient did not want chemotherapy and because she had a high Ki-67 index, corticosteroid treatment was started. This approach may be justified because patients who are CD56 – carry a good prognosis.

## Conclusion

SPTCL is a rare entity; only a very few cases have been reported from India.[7] SPTCL is characterized by the rimming of fat spaces by T cells showing a degree of atypia.[8] Meticulous examinations of slides are essential in a case of panniculitis as the diagnosis of SPTCL can be easily missed if this possibility is not included in the differential diagnosis of patients with panniculitis.
